# Concurrence of seizures and peri-ictal delirium in the critically ill - its frequency, associated characteristics, and outcomes

**DOI:** 10.1007/s00415-023-11944-3

**Published:** 2023-09-07

**Authors:** Anja I. Frei, Anna S. Wagner, Sira M. Baumann, Pascale Grzonka, Sebastian Berger, Sabina Hunziker, Stephan Rüegg, Stephan Marsch, Raoul Sutter

**Affiliations:** 1 Department of Intensive Care, University Hospital Basel, Basel, Switzerland; 2 Department of Neurology, University Hospital Basel, Basel, Switzerland; 3 Medical Faculty, University of Basel, Basel, Switzerland; 4 Department of Psychosomatic Medicine, University Hospital Basel, Basel, Switzerland

**Keywords:** Seizures, Delirium, Intensive care units, Neurocritical care

## Abstract

**Background:**

To assess the frequency, clinical features, and outcome of peri-ictal delirium in adult patients experiencing seizures during intensive care.

**Methods:**

This observational study was conducted at a Swiss intensive care unit from 2015 to 2020. Patients aged ≥ 18 years with seizures were categorized as peri-ictal delirious (Intensive Care Delirium Screening Checklist [i.e., ICDSC] ≥ 4) or not (i.e., ICDSC < 4) within 24 h of seizures. The frequency of peri-ictal delirium and in-hospital death were defined as the primary endpoints. Illness severity and treatment characteristics between delirious and non-delirious patients were secondary endpoints. Logistic regression was used to compare in-hospital death and differences regarding clinical characteristics between delirious and non-delirious patients.

**Results:**

48% of 200 patients had peri-ictal delirium for a median of 3 days. Delirious patients were older (median age 69 vs. 62 years, *p* = 0.002), had lower Simplified Acute Physiology Scores II (SAPS II; median 43 vs. 54, *p* = 0.013), received neuroleptics more frequently (31 vs. 5%, *p* < 0.001), were mechanically ventilated less often (56% vs. 73%, *p* = 0.013) and shorter (median 3 vs. 5 days, *p* = 0.011), and had decreased odds for in-hospital death with delirium (OR = 0.41, 95% CI 0.20–0.84) in multivariable analyses.

**Conclusions:**

Delirium emerged in every second patient experiencing seizures and was associated with lower SAPS II, shorter mechanical ventilation, and better outcomes, contradicting assumptions that altered cerebral function, from seizures and delirium, are linked to unfavorable outcomes.

## Introduction

Several studies have investigated the emergence of transient cerebral dysfunction in critically ill patients admitted to intensive care units (ICUs) with frequent clinical manifestations, such as delirium [1, 7–9, 13, 22], and less frequently reported complications, such as seizures [4, 8, 31]. The term delirium is defined as a complex syndrome with a varying duration and combining at least four well-defined clinical symptoms including symptom fluctuation, disturbed sleep/wake-cycles, inappropriate speech or mood, psychomotor agitation or retardation, hallucinations/delusions/psychosis, disorientation, inattention, and altered consciousness [5]. In contrast, seizures are a sudden and temporary disturbance in the electrical activity of the brain, which can cause changes in behavior, sensation, motor activity or consciousness, lasting less than 5 min. They are often accompanied by focal or bilateral motor symptoms typically involving body parts according to their representation on the hyperactive motor cortex as outlined in the diagnostic manual of the International League Against Epilepsy (ILAE).

Despite their different clinical manifestation and frequency, both appear to be associated with increased morbidity, prolonged and intensified treatment, and adverse outcomes. However, the concurrent presentation of these neurological complications in critically ill patients remains inadequately explored, and there is a lack of understanding of the associated clinical characteristics and specific outcomes.

We, therefore, aimed to assess the frequency, clinical features and associated short-term outcome of peri-ictal delirium in critically ill patients experiencing seizures during intensive care.

## Methods

### Setting, study design and ethics

In the current study, we use data from a previously registered cohort study conducted at the ICU of the University Hospital of Basel, a Swiss academic tertiary medical center (NCT03860467; https://classic.clinicaltrials.gov/ct2/show/NCT03860467). The study was granted approval by the local ethics committee (Ethikkommission Nordwest- und Zentralschweiz), in accordance with the ethical principles outlined in the 1964 Declaration of Helsinki, along with its subsequent revisions. As per the committee's ethical evaluation (EKNZ No. 2018-02361), the requirement for patients’ informed consent was waived. To ensure the quality and uniformity in the reporting of observational studies, the STROBE guidelines were adhered to [29].

### Data collection

All adult (≥ 18 years of age) ICU patients from January 1st 2015 to December 31st 2020 with reported isolated seizures were retrospectively assessed as previously reported (details regarding the definition of seizures outlined below) [30, 31]. Patients with persistent seizures fulfilling the criteria of status epilepticus (SE) were excluded. The prospectively recorded digital electroencephalographic (EEG) and ICU information system MetaVision (iMDsoft, Wakefield, MA) databases were screened to retrospectively collect and enter the following data into a predefined case report form: demographics, history of previous seizures and/or delirium, presumed etiology of seizures categorized as potentially non-fatal or fatal as previously defined [21, 30, 31], the Charlson Comorbidity Index [6], and the Simplified Acute Physiology Score II (SAPS II) [18]. In addition, several markers of systemic inflammation, including maximal core body temperature, serum concentrations of C-reactive protein (CRP) expressed in mg per liter, and white blood cell counts were assessed. Furthermore, the Glasgow Coma Score (GCS) [17] documented at seizure onset, seizure semiology (if reported, including single or repetitive seizures), and seizure evolution (defined as focal, focal to bilateral or primarily bilateral) were recorded. In addition, delirium-associated symptoms as assessed by the Intensive Care Delirium Screening Checklist (ICDSC) [5] with the highest ICDSC score on the day of seizure onset and 24 h prior and after seizure were assessed, as well as the emergence of delirium within this timeframe defined as an ICDSC ≥ 4 (details regarding ICDSC assessment outlined below).

Treatment parameters that were evaluated included the length of sedation and mechanical ventilation, the number of inserted drainages and catheters, administration of antipsychotic (neuroleptic) and antiseizure medications, as well as the duration of in-hospital treatment and ICU stay. Additionally, complications that emerged during intensive medical care were documented, such as organ failure and infections identified within a 7-day period preceding the onset of seizures. Infections were detected using the protocol outlined in earlier investigations [3, 24, 27], in accordance to the guidelines published by the Centers for Disease Control and Prevention (CDC) [11]. Outcome at hospital discharge, such as return to premorbid neurologic function and death, were assessed.

### Definition of seizures

As in our previous study [31], patients were categorized as having seizures using predefined criteria. The diagnosis of seizures depended on the additional following three point: (1) patients needed to show improvement in consciousness and/or neurologic function after their seizure has been observed while in the intensive care unit; (2) the patients’ EEGs following seizures had to show evidence of repeated epileptiform discharges, such as spikes and/or sharp waves, following the first seizure; (3) or seizures had to be detected by EEG. In addition, patients with motor symptoms had to exhibit motor symptoms that align with typical seizures, as outlined by the ILAE. In accordance with the ILAE (diagnostic manual) motor symptoms had to be focal or bilateral and typically involve body parts linked to their representation on the motor cortex. These symptoms may include rhythmic myocloni, Jacksonian march, tonic muscle contractions, mutual contractions of agonist and antagonist muscles producing athetosis or twists, contraction clusters for milliseconds (jerks), sudden loss or diminution of muscle tone, flexion, extension or both for seconds (spasms) in series involving proximal and truncal muscles, hyperkinetic movements such as pedaling, jumping, pelvic thrusting, thrashing and/or rocking movements, automatisms defined as repetitive movements resembling voluntary action but undertaken without volition, dysarthria/anarthria while language functions remain intact, and forced conjugate ocular, cephalic, and/or truncal version, rotation, or lateral deviation (left or right).

All patients who exhibited clinical, electrographic, or electro-clinical seizures with incomplete recovery of neurologic function and consciousness within 30 min following the seizure received EEGs. Two trained and board-certified EEG specialists visually evaluated all EEGs, and in cases of disagreement, consensus was reached through additional joint review.

### Definition and screening of delirium

As described in our previous study on postictal delirium following SE [4], in our institution, the ICDSC was routinely used to screen patients for the emergence of delirium. According to the studies and guidelines mentioned above, an ICDSC ≥ 4 was defined as delirium [2, 5]. An ICDSC ≥ 4 documented prior to seizure onset was defined as pre-ictal delirium, an ICDSC ≥ 4 in the aftermath of seizures as post-ictal delirium. Specialized nurses conducted systematic screenings using the ICDSC every 8 h throughout the entire study duration. First, the nurses assessed alterations in consciousness from baseline, inattention, disorganized thinking, and hallucinations or delusions. In the subsequent step, psychomotor activity (agitation or retardation), speech/mood, sleep–wake cycle, and symptom fluctuations were evaluated over the same duration of the 8-h shift. Patients’ Richmond Agitation-Sedation Scale (RASS) was also assessed every 8 h, ranging from − 5 (deep coma) to + 4 (combative) [10]. The ICDSC was evaluated once patients had recovered to a RASS level greater than − 4. In patients with delirium, the screening for ICDSC was expanded in both directions, the time prior and after the 24 h of seizure onset until ICDSC was < 4 to calculate the overall duration of delirium.

### Outcomes

The frequency of peri-ictal delirium among critically ill patients experiencing seizures and in-hospital death were defined as the primary endpoints and differences regarding demographics, illness severity, and treatment characteristics between delirious and non-delirious patients were secondary endpoints.

### Statistics

Patients were first categorized into patients with seizures during their ICU stay with and without peri-ictal delirium. Further, patients were categorized as patients with and without in-hospital death. Univariable comparisons of proportions were performed between the two groups of each categorization using Chi-square and Fisher exact test (where appropriate). Continuous variables were compared using the Student *t* test for normal distributions and the Mann–Whitney* U* test for non-normal distributions. Categorical variables were presented as counts (percentage), while continuous variables were expressed as medians and interquartile ranges (IQRs). Demographics, clinical, and treatment-related variables with significant differences among these groups were included in the uni- and multivariable logistic regression analyses to calculate the odds for each variable for in-hospital death. The final multivariable logistic regression model was assessed for goodness-of-fit using the Hosmer–Lemeshow *χ*^2^ test, which compares observed and estimated outcomes and provides measures of calibration [15]. Given that delirium occurring before (i.e., pre-ictal) and after (i.e., post-ictal) seizures could potentially signify distinct clinical entities and exhibit varying associations with specific outcomes, sensitivity analyses were conducted to evaluate the relationship between each of these two entities and outcome measures.

A two-tailed *p* value of ≤ 0.05 was considered statistically significant. All statistical analyses were performed using STATA^®^16.1 (Stata Corp., College Station, TX, USA).

## Results

**Fig. 1:**
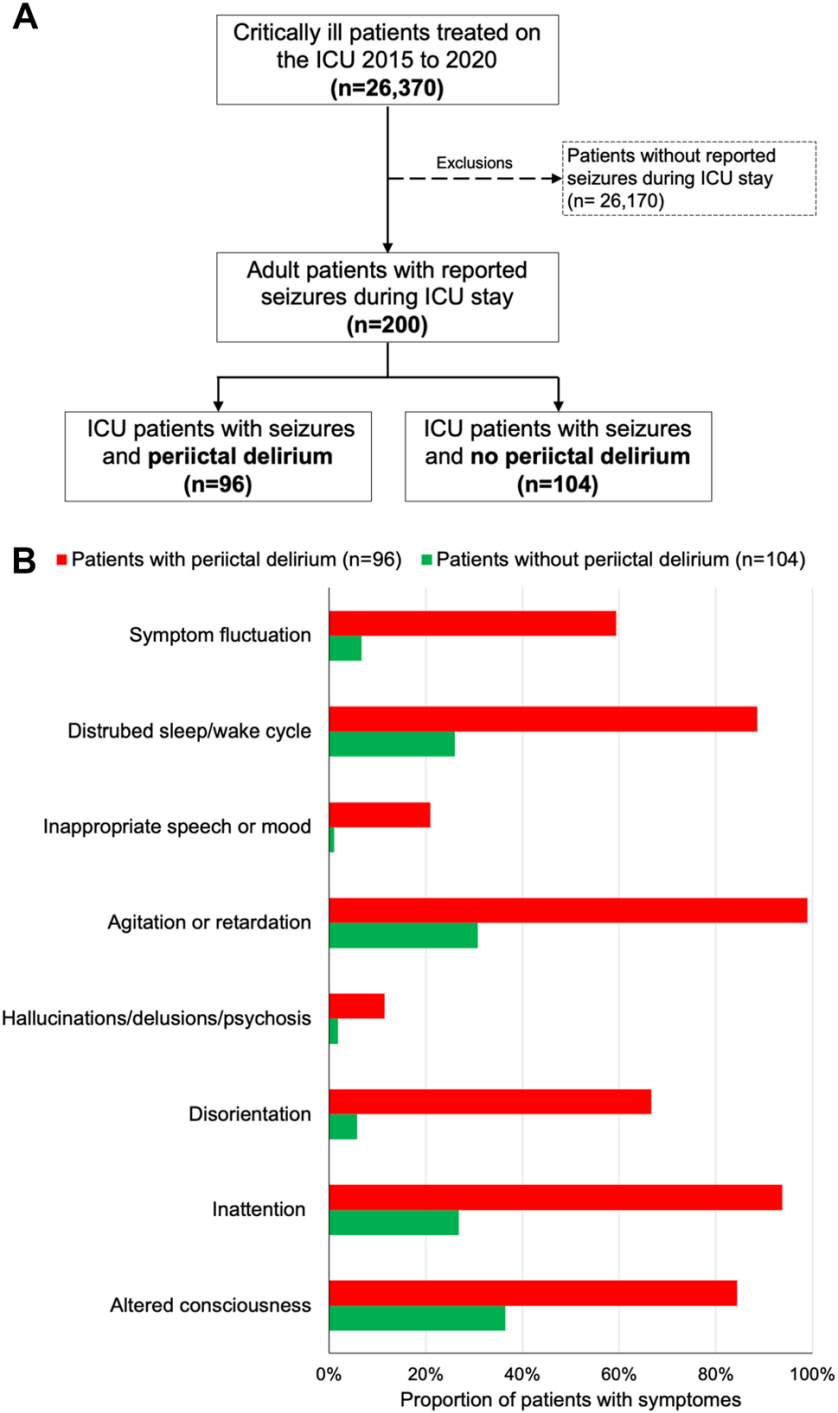
Flow chart (**A**) and proportion of delirium associated symptoms as assessed by the ICDSC in seizing patients with and without peri-ictal delirium (**B**). *ICU* intensive care unit, *ICDSC* Intensive Care Delirium Screening Checklist

Among 26,370 critically ill patients treated in the intensive care unit from 2015 to 2020, 200 patients (0.76%) were identified as having seizures during intensive care. Of those, 96 (48%) were delirious 24 h around seizure onset according to the ICDSC (Fig. 1A), with a median duration of delirium of 3 days. Post-ictal delirium was more common than pre-ictal delirium (64.6% versus 35.4%). Differences regarding symptoms in patients with and without peri-ictal delirium as assessed by the ICDSC are outlined in Fig. 1B**,** with disturbed sleep/wake cycle, psychomotor agitation or retardation, inattention, and altered consciousness being the most frequent symptoms in patients with peri-ictal delirium.

### Univariable comparisons between delirious and non-delirious patients

Univariable comparisons of clinical characteristics known on the day of seizure onset between patients with and without concomitant peri-ictal delirium are presented in Table 1. Delirious patients were older and had a lower median Simplified Acute Physiology Score II. History of seizures and previous delirium, as well as seizure type (i.e., focal or bilateral, and isolated or repetitive) did not differ between delirious and non-delirious patients. Table 2 presents the univariable comparison of treatment characteristics, complications, and short-term (in-hospital) outcomes between patients with and without concomitant peri-ictal delirium. As compared to non-delirious patients, delirious patients received neuroleptic drugs more often following the detection of delirium, were mechanically ventilated less frequently and for a shorter median duration, had a longer median in-hospital treatment, and died less often during their hospital stay. Analyses after excluding non-survivors, however, revealed no differences regarding median in-hospital stay. Other treatment characteristics, such as the number of inserted catheters and drainages, number of antiseizure drugs, length of ICU-stay, and complications reported during intensive care did not differ significantly.

**Table 1:** Univariable comparisons of clinical characteristics of patients with seizures with and without peri-ictal delirium treated in the intensive care unit (*n* = 200)

	Patients with delirium (ICDSC ≥ 4; *n* = 96)		Patients without delirium (ICDSC < 4; *n* = 104)		*p* value
	*n*/median	%/IQR	*n*/median	%/IQR	
Demographics and admission characteristics					
Age (years; median, IQR)	68.5	59.5–76.5	61.5	48.5–72.0	**0.001**
Female (*n*, %)	45	46.9	43	41.4	0.431
Male (*n*, %)	51	53.1	61	58.6	
Potentially fatal etiology (*n*, %)	74	77.1	87	83.7	0.241
Patient history (*n*, %)					
History of proceeding seizure or epilepsy	45	46.9	41	39.4	0.288
History of previous delirium	5	5.2	2	1.9	0.266
History of alcohol abuse/withdrawal	11	11.5	8	7.7	0.470
Illness severity scoring at seizure onset (median, IQR)					
Lowest GCS	7	3–11	3	3–8	**0.002**
SAPS II	43	30–60	53.5	41–64	**0.013**
Charlson Comorbidity Index	5	3–7	4	2–6	0.061
Delirium features					
Number of patients with delirium on day before seizure (*n*, %)	34	35.4			
Number of patients with delirium on seizure day (*n*, %)	61	63.5			
Number of patients with delirium on day after seizure (*n*, %)	62	64.5			
Delirium on day of ICU admission (*n*, %)	7	3.5			
Duration of delirium (days; median, IQR)	3	2–7			
Maximal ICDSC (median, IQR)	5	4–6	0	0–2.5	** < 0.001**
ICDSC 24 h before seizure (median, IQR)	1	0–4.5	0	0–0.5	** < 0.001**
ICDSC on day of seizure (median, IQR)	4	2–5	0	0–0.5	** < 0.001**
ICDSC 24 h after seizure (median, IQR)	4	2.5–5	0	0–0.5	** < 0.001**
Parameters of inflammation					
CRP on day before seizure (mg/l; median, IQR)	32.6	4.1–107.5	30	6.1–63.5	0.464
CRP on day on seizure day (mg/l; median, IQR)	29.6	5.2–95.7	24	3.6–88.6	0.481
CRP on day after seizure in mg/l (mg/l; median, IQR)	45.7	17.5–100.7	42.9	14.8–150.7	0.676
Leukocytes on day before (× 10^9^/l; median, IQR)	9.7	7.3–11.2	10.7	8.6–14.4	**0.047**
Leukocytes on seizure day (× 10^9^/l; median, IQR)	9.7	7.9–11.6	10.0	7.3–14.4	0.458
Leukocytes on day after seizure (× 10^9^/l; median, IQR)	9.2	7.3–11.2	10.4	8.0–13.0	**0.031**
Temperature on day before seizure (°C; median, IQR)	37.2	36.8–37.9	37.4	36.8–38.0	0.888
Temperature on seizure day (°C; median, IQR)	37.4	36.9–37.9	37.3	36.9–37.8	0.986
Temperature on day after seizure (°C; median, IQR)	37.4	37.1–37.7	37.5	37.1–37.8	0.376
Seizure characteristics (*n*, %)					
Repetitive seizures (*n*, %)	69	71.9	72	69.2	0.682
Focal seizures (*n*, %)	33	34.4	33	31.7	0.691
Bilateral seizures (*n*, %)	44	45.8	54	51.9	0.389
Unknown seizure type (*n*, %)	19	19.8	17	16.4	
Performed EEG (*n*, %)	86	89.6	86	82.7	0.221

Bold font indicates statistical significance set at a *p* < 0.05

*IQR* interquartile range, *SAPS II* Simplified Acute Physiology Score II (range 0–163) [18], *ICDSC* Intensive Care Delirium Screening Checklist (range 0–8) [5], *GCS* Glasgow Coma Score (range 3–15) [17], *CRP* C-reactive protein, *EEG* electroencephalography

**Table 2:** Univariable comparisons of treatment, complications and outcomes of patients with seizures with and without peri-ictal delirium treated in the intensive care unit (*n* = 200)

	Patients with delirium (ICDSC ≥ 4; *n* = 96)		Patients without delirium (ICDSC < 4; *n* = 104)		*p* value
	*n*/median	%/IQR	*n*/median	%/IQR	
Treatment characteristics					
In-hospital stay (days; median, IQR)	17	10–27	14	7–23	**0.050**
In-hospital stay in survivors (days; median, IQR)	17	10–32	16	10–26	0.385
ICU stay (days; median, IQR)	6	3–10	5	2–11	0.449
ICU stay in survivors (days; median, IQR)	6	3–10	5	1–12	0.468
Sedation and mechanical ventilation during seizure (*n*, %)	54	56.3	76	73.1	**0.013**
Duration of mechanical ventilation (in intubated patients in days; median, IQR)	3	1–7	5	2–9	**0.011**
Duration of mechanical ventilation in survivors (in intubated patients in days; median, IQR)	2	1–6	7	2–9	**0.005**
Use of benzodiazepines for seizure (*n*, %)	51	53.1	53	51.0	0.760
Administration of additional antiseizure drugs following seizure (*n*, %)	71	74.0	73	70.2	0.553
Number of additional antiseizure drugs following seizure (median, IQR)	1	0–1	1	0–2	0.989
Administration of antipsychotic drugs (*n*, %)	31	32.3	5	4.8	** < 0.001**
Number of catheters and drainages (median, IQR)	5	3–6	5	4–6	0.252
Complications (*n*, %)					
Renal failure at seizure onset	30	31.3	22	21.2	0.104
Liver failure at seizure onset	4	4.2	8	7.7	0.378
Multiorgan failure at seizure onset	5	5.2	5	4.8	1.000
Infections	39	40.6	46	44.2	0.668
Ventilator associated pneumonia	16	16.7	20	19.2	0.637
Outcomes (*n*, %)					
Return to premorbid neurologic function at discharge	17	17.7	17	16.4	0.798
Death at hospital discharge	13	13.5	29	27.9	**0.013**

Bold font indicates statistical significance set at a *p* < 0.05

*IQR* interquartile range, *ICDSC* Intensive Care Delirium Screening Checklist (range 0–8) [5], *ICU* intensive care unit

### Uni- and multivariable comparisons between patients with and without in-hospital death

Table 3 presents univariable comparisons of patient-related clinical characteristics known at seizure onset between survivors (158; 79%) and non-survivors (42; 21%). Non-survivors were older, less commonly delirious, reached a lower level of consciousness on the day of seizure, had a higher SAPS II, and potentially fatal etiologies of seizures were more frequent. However, seizure characteristics did not differ significantly. Late complications in the course of intensive care were more frequent in non-survivors (renal failure 43% vs. 22%, *p* = 0.005; liver failure 19% vs. 3%, *p* = 0.001; multiorgan failure 21% vs. 1%, *p* < 0.001).

**Table 3:** Univariable comparisons of patients related characteristics at seizure onset between patients with seizures with and without in-hospital death (*n* = 200)

	Patients with in-hospital death (*n* = 42)		Patients without in-hospital death (*n* = 158)		*p* value
	*n*/median	%/IQR	*n*/median	%/IQR	
Demographics and admission characteristics					
Age (years; median, IQR)	70	65–79	62.5	50–73	**0.002**
Female (*n*, %)	19	45.2	69	43.7	0.856
Male (*n*, %)	23	54.8	89	56.3	
Potentially fatal etiology (*n*, %)	40	95.2	121	76.6	**0.004**
Illness severity scoring at seizure onset (median, IQR)					
Lowest GCS on day of seizure	3	3–6	6	3–11	**0.005**
SAPS II	61.5	51–71	47	30–59	** < 0.001**
Charlson Comorbidity Index	6	4–7	4	3–7	0.066
Seizure characteristics (*n*, %)					
Repetitive seizures (*n*, %)	29	69.1	112	70.9	0.816
Focal seizures (*n*, %)	11	26.2	55	34.8	0.291
Bilateral seizures (*n*, %)	25	59.5	73	46.2	0.125
Unknown seizure type (*n*, %)	6	14.3	30	19.0	0.481

Bold font indicates statistical significance set at a *p* < 0.05

*IQR* interquartile range, *SAPS II* Simplified Acute Physiology Score II (range 0–163) [18], *ICDSC* intensive Care Delirium Screening Checklist (range 0–8) [5], *GCS* Glasgow Coma Score (range 3–15) [17], *CRP* C-reactive protein

Uni- and multivariable logistic regression analyses including all clinical variables known at seizure onset are presented in Table 4. Multivariable analyses revealed peri-ictal delirium to be associated with decreased odds for in-hospital death independent of other potential confounders, including increasing age, potentially fatal etiologies, increasing level of consciousness on the day of seizure, and decreasing SAPS II. The Hosmer Lemeshow goodness-of-fit test revealed insignificant *p* values representing adequate model fit (*χ*^2^ 7.45, *p* = 0.489) indicating an adequate fit of the performed multivariable model.

**Table 4:** Uni- and multivariable logistic regression analysis regarding potential associations of in-hospital death known at seizure onset in patients with and without delirium

Potential associations of in-hospital death (present at seizure onset)	Univariable			Multivariable		
Including patients with coma at seizure onset	OR	95% CI	*p* value	OR	95% CI	*p* value*
Age (per every additional year)	1.04	1.01–1.06	**0.004**	1.03	0.99–1.06	0.112
Potentially fatal etiology	6.12	1.41–20.52	**0.016**	4.77	1.01–19.42	**0.048**
GCS at seizure onset (per increasing unit)	0.85	0.77–0.95	**0.004**	1.08	0.92–1.27	0.338
SAPS II at seizure onset (per increasing unit)	1.06	1.04–1.09	** < 0.001**	1.06	1.02–1.11	**0.002**
Peri-ictal delirium	0.41	0.20–0.84	**0.015**	0.42	0.18–0.96	**0.040**

Bold font indicates statistical significance set at a *p* < 0.05

*OR* odds ratio, *CI* confidence interval

*Insignificant Hosmer–Lemeshow goodness-of-fit test indicating adequate model fit (*χ*^2^ 7.45, *p* = 0.489)

### Sensitivity analyses regarding pre- and postictal delirium

Given that delirium occurring before (i.e., pre-ictal) and after (i.e., post-ictal) seizures might signify distinct clinical entities and exhibit varying associations with specific outcomes, sensitivity analyses were conducted to evaluate the relationship between each of these two entities and in-hospital death. Such analyses revealed insignificant associations either for increased or decreased odds regarding in-hospital death for the multivariable models adjusting for the same potential confounders (for pre-ictal delirium: adjusted OR_for death_ 1.52; 95% CI 0.50–4.64; for post-ictal delirium: adjusted OR_for death_ 0.46, 95% CI 0.18–1.18).

## Discussion

Our investigation reveals that peri-ictal delirium manifests in nearly half of the critically ill patients who suffer from seizures while in intensive care, with post-ictal delirium being more frequent than pre-ictal delirium. It is commonly assumed that manifestations of altered cerebral function, as mirrored by seizures and delirium, are closely linked to unfavorable prognoses and outcomes in critically ill patients [1, 4, 9, 13, 22, 31]. However, our study of a severely ill patient cohort with seizures demonstrates that delirium is associated with lower illness severity, as quantified by the SAPS II. Surprisingly, our study further suggests that patients with peri-ictal delirium experience shorter periods of mechanical ventilation and die less frequently during their hospital stay. As patients who died during their hospital stay may have died relatively early during the course of disease, subsequent analyses regarding duration of ICU and hospital stay were performed after excluding non-survivors. These analyses revealed no significant differences between delirious and non-delirious patients regarding ICU and hospital stay. However, mechanical ventilation remained significantly shorter in delirious patients. These findings and especially the shorter mechanical ventilation of delirious patients contradict other studies who suggest an association of ICU delirium and prolonged mechanical ventilation as well as increased mortality [19]. Multivariable analyses indicate that, independent of potential confounders identified in our univariable comparisons, patients with peri-ictal delirium have low odds of short-term in-hospital death. Although sensitivity analyses for both pre- and post-ictal delirium did not observe a consistent and significant reduction in the odds of in-hospital mortality, an increased risk of delirium with such adverse short-term outcomes that would have been in line with the current assumptions was not found in any of our analyses.

As the current evidence from the literature clearly indicates that delirium emerging as a complication during intensive care promotes increased mortality rather than survival [16], the findings of our study call for an explanation. As in our institution patients are not routinely sent to the ICU because of single seizures not transforming into status epilepticus, a selection towards less critically ill patients, for example, patients with uncontrolled epilepsy but no other critical illnesses can be excluded. However, the fact that non-delirious patients had a higher SAPS II indicating being more critically ill as compared to delirious patients suggests a potential underlying selection bias towards less critically ill patients. As in contrast to single seizures, delirium is a criterion to be admitted to the ICU, especially if the intermediate care unit has no capacity, the admission of delirious but otherwise not critically ill patients may be a possible explanation. However, as only 7 patients (3.5% of our cohort) were delirious on the day of admission to the ICU, it is unlikely that admission for treatment of delirium is the only explanation for such potential self-fulfilling prophecy. Another possible explanation that cannot be excluded from our study may be that our study population was not representative of all critically ill patients with delirium.

The high proportion of critically ill patients with delirium in close temporal relation to seizures seems surprising at first glance. However, it appears to be in line with several prior studies on delirium following SE [3, 4] and studies on delirium in critically ill populations without epileptic complications [8, 12, 14] but higher than in mixed cohorts of neurocritically ill patients with delirium in 12–43% [20] and 22% in post-surgery ICU patients [23]. The larger proportion of patients with delirium emerging in the post-ictal phase compared to delirium evolving hours before registered seizure onset suggests that seizures in critically ill patients may be important promotors for delirium, a hypothesis that calls for further studies.

When accounting for previous reports of “post-ictal encephalopathies”, the coincidence of delirium with seizures seems less surprising. However, it is crucial to note that the terms “post-ictal encephalopathy” and “delirium” should not be used interchangeably, even though distinguishing between the two may be challenging. Manifestation of altered consciousness, disorientation, and/or inattention in “post-ictal encephalopathies” cannot be equated with delirium, the latter requiring a more complex combination of at least four well-defined symptoms, as outlined in the ICDSC [5]. These insights prompted ten societies to issue an extensive statement regarding the nomenclature of delirium and encephalopathy, recommending that delirium be regarded as a subcategory of encephalopathy [25].

The fact that none of the investigated inflammatory parameters were associated with the occurrence of seizures and delirium, and on the contrary, the leukocyte count in delirious patients was lower than in non-delirious patients, may seem surprising, since in earlier studies, systemic inflammation or infections in SE patients were associated with a worse disease course and outcome [24, 26–28, 33]. However, the serum concentrations of CRP in our cohort increased with every day of observation. Possible explanations for not reaching significance are the limited sample size and the relatively short time of observation.

With a median of 3 days, the duration of delirium in our cohort was slightly longer as compared to delirium seen in patients following SE [3, 4]. However, a finding common to both patient with isolated seizures and SE is delirium emerging more frequently in patients with a higher level of consciousness at seizure onset. One possible albeit daring interpretation of this finding is that a higher level of consciousness at seizure onset and the occurrence of delirium may signify the preservation of complex neuronal functions in the central nervous system, a notion that warrants further exploration. While our results do not imply that the occurrence of delirium in critically ill patients with seizures is a reliable indicator of improved outcomes, they do challenge the prevailing notion that multiple symptoms of impaired brain function are associated with a faster disease progression or poor outcomes. In the absence of compelling evidence to the contrary, clinicians are urged to refrain from embracing the conventional assumption that an unfavorable prognosis is probable for this patient cohort.

### Strengths and limitations

The strength of our study is the relatively large cohort, the observation period of 5 years at a tertiary academic medical care center, and the use of comprehensive prospectively monitored clinical data during the entire study period with the digital ICU information system MetaVision (iMDsoft, Wakefield, MA).

It is important to note that our study was designed as a single-center observational study, which may limit the generalizability of our findings. Nonetheless, the data we analyzed were systematically and prospectively recorded in a digital ICU information system, and was available for all patients, thereby reducing the possibility of selection bias. Additionally, the routine clinical practice in the ICU ensured the systematic collection of clinical data related to treatment, monitoring measures, and complications. Moreover, the standardized diagnostic procedures and management of seizures and delirium were performed by trained and specialized nurses and a consulting team of neurologists and neurocritical care specialists, which remained consistent throughout the entire study period. Another limitation is the fact that by the retrospective nature of the study, epileptic seizures that may have not been clinically overt may have been missed and are underrepresented by our cohort.

A similar limitation comes from the retrospective assessment of reported seizure semiology, which was lacking in some patients. Moreover, very short-lasting delirium might have been missed since the ICDSC was performed every eight hours only. This time span, however, reflects the daily clinical practice of many ICUs, as more frequent ICDSC assessments would be very labor intensive and interfere with patient care if performed by trained nurses. However, such screening every eight hours may be especially critical, as mental status of our patients may alter frequently in the post-ictal period. These limitations are likely to have affected the temporal allocation of delirium and seizures when applying our 24 h window. Moreover, the retrospective study design lacked the ability to discriminate between catatonia and delirium [32], which plausibly inflated the incidence of delirium. Additionally, the extent to which a “novel baseline neurologic function” manifested after seizures could not be evaluated, conceivably leading to an overestimation of the occurrence of post-ictal delirium. Finally and as discussed above, the retrospective nature of our study does not exclude the possibility of an underlying and undetected selection bias that may explain why delirious patients in our cohort were less severely ill. This, however, hardly detracts from the conclusion of our study that the co-occurrence of epileptic seizures and delirium can not necessarily be equated with a poor prognosis of ICU patients.

## Conclusions

Peri-ictal delirium appears to be a common complication among patients experiencing seizures during intensive care, with delirium occurring in nearly every second patient and post-ictal delirium being more frequent than pre-ictal delirium. Interestingly, our observations suggest that peri-ictal delirium is associated with a decreased SAPS II, shorter mechanical ventilation, and better outcomes in our patient cohort. The observations made in this study challenge the prevalent assumption that altered cerebral function, as evidenced by seizures and delirium, is a harbinger of unfavorable outcomes. Further comprehensive investigations are required.

## Data Availability

The corresponding author has full access to all of the data in the study. He takes full responsibility for the integrity of the data, the accuracy of the data analysis and interpretation, and the conduct of the research. The authors have the right to publish any and all data, separate and apart from the guidance of any sponsor.
